# Outcomes following kidney transplantation in patients with sickle cell disease: The impact of automated exchange blood transfusion

**DOI:** 10.1371/journal.pone.0236998

**Published:** 2020-08-13

**Authors:** Joanna C. Willis, Moji Awogbade, Jo Howard, Cormac Breen, Allifia Abbas, Mark Harber, Ali M. Shendi, Peter A. Andrews, Jack Galliford, Raj Thuraisingham, Alice Gage, Sapna Shah, Claire C. Sharpe

**Affiliations:** 1 Renal Medicine, King’s College Hospital NHS Foundation Trust, London, United Kingdom; 2 Haematological Medicine, King’s College Hospital NHS Foundation Trust, London, United Kingdom; 3 Haematology, Guy’s and St Thomas’ NHS Foundation Trust, London, United Kingdom; 4 Nephrology and Transplantation, Guy’s and St Thomas’ NHS Foundation Trust, London, United Kingdom; 5 Renal Medicine, Royal Free London NHS Foundation Trust, London, United Kingdom; 6 Renal Medicine, Zagazig University, Zagazig, Egypt; 7 Renal Medicine, Epsom and St Helier University Hospitals NHS Trust, London, United Kingdom; 8 Nephrology, Imperial College Healthcare NHS Trust, London, United Kingdom; 9 Department of Renal Medicine and Transplantation, Barts Health NHS Trust, London, United Kingdom; 10 Department of Inflammation Biology, King’s College London, London, United Kingdom; Medical University of Gdansk, POLAND

## Abstract

There are over 12,000 people with sickle cell disease (SCD) in the UK, and 4–12% of patients who develop Sickle Cell Nephropathy (SCN) progress to End Stage Renal Disease (ESRD). Renal transplantation offers the best outcomes for these patients with but their access to transplantation is often limited. Regular automated exchange blood transfusions (EBT) reduce the complications of SCD and may improve outcomes. However, concerns over alloimmunisation limit its widespread implementation. In this retrospective multicenter study, data were collected on 34 SCD patients who received a kidney transplant across 6 London Hospitals between 1997 and 2017. 20/34 patients were on an EBT program, pre or post renal transplantation. Overall patient and graft survival were inferior to contemporaneous UK data in the ESRD population as a whole, a finding which is well-recognised. However, patient survival (CI 95%, p = 0.0032), graft survival and graft function were superior at all time-points in those who received EBT *versus* those who did not. 4/20 patients (20%) on EBT developed *de novo* donor specific antibodies (DSAs). 3/14 patients (21%) not on EBT developed *de novo* DSAs. The incidence of rejection in those on EBT was 5/18 (28%), as compared with 7/13 (54%) not on EBT. In conclusion, our data, while limited by an inevitably small sample size and differences in the date of transplantation, do suggest that long-term automated EBT post renal transplant is effective and safe, with improvement in graft and patient outcomes and no increase in antibody formation or graft rejection.

## Introduction

Sickle cell disease (SCD) is endemic in sub-Saharan Africa, India, Saudi Arabia and the Mediterranean. However, as a result of migration it is becoming increasingly prevalent in other parts of the world and, in high income settings, survival into adulthood has consistently increased. In the UK, it is estimated at least 12,000 people are living with the disease [1], with 99% surviving into adulthood [2]. Chronic kidney disease (CKD) secondary to sickle cell nephropathy (SCN) is becoming more prevalent as the life expectancy of patients with SCD improves [3, 4]. Microalbuminuria, an early manifestation of SCN, reaches a prevalence of approximately 60% in those over 45 years [5], and although only 4–12% of patients with SCD are reported to develop end-stage renal disease (ESRD) [6], CKD was reported as the cause of death in 45% of patients over 60 in a Jamaican cohort [7].

Outcome data for patients with SCD on renal replacement therapy (RRT) are few but dialysis dependency is associated with a very poor prognosis. A five-year study of patients with SCD receiving hemodialysis in France reported a 7-fold increase in the risk of death for these patients compared to patients without SCD, and a much lower incidence of renal transplantation (26 vs 54%), thought to be due to a combination of poorer cardiovascular fitness, ethnicity and sensitization [8]. Despite this, kidney transplantation offers the best outcome for patients with SCD and ESRD. Although long-term graft and patient survival in SCD are inferior compared to patients with other causes of ESRD, the prognosis is far better after transplantation compared to receiving dialysis and is now similar to that of patients with diabetes [9, 10].

Blood transfusions are an established treatment for the management of both acute and chronic complications of SCD and are routinely used for stroke prevention, acute chest crisis and multi-organ failure. However, there is little evidence for the benefits of regular blood transfusion for the prevention of renal complications, and none following renal transplantation [11].

Blood transfusion is generally avoided in patients being considered for renal transplantation due to the risks of HLA sensitization but total avoidance is extremely difficult in patients with SCD and ESRD due to severe anaemia [11, 12]. Transplant surgery on severely anemic patients with a high percentage of sickle hemoglobin is high risk, potentially triggering life- and allograft-threatening vaso-occlusive complications, with an increased risk of delayed graft function or primary non-function [13].

Blood transfusion in SCD can be given as a simple top-up or as an (automated or manual) exchange blood transfusion (EBT) involving the simultaneous or sequential removal of red cells and replacement with donor red cells. The advantages of automated red cell exchange over top-up transfusion are improved reduction of hemoglobin S (HbS), longer intervals between transfusions and reduced iron loading.

The aim of this study was to report the outcomes of patients with sickle cell disease undergoing renal transplantation and to analyze the impact of exchange blood transfusion therapy on outcomes.

## Materials and methods

In this retrospective multicenter study, data were collected on SCD patients transplanted and followed up over a 20-year period at 6 London Renal Units between January 1997 and January 2017 (see S1 File). The diagnosis of SCD was confirmed in each case by haemoglobin electrophoresis. All transplanted organs were sourced either through UK NHS Blood and Transplant’s deceased donor pool or through Live donation. None of the transplant donors was from a vulnerable population and all donors or next of kin provided written informed consent that was freely given through a standardized NHS Blood and transplant consent process, embedded in UK legislation. The study was reviewed by the internal Research and Innovations Office and was not considered to need research ethics committee approval and so was registered on the King’s College Hospital NHS Foundation Trust Nephrology Audit Register as audit number REN015.

Patient and graft survival, graft function, incidence of biopsy-proven rejection and markers of HLA sensitization were the main outcome measures, and these outcomes were first assessed in the cohort as a whole, and then compared between those patients on EBT programmes and those not on EBT programmes.

Estimated glomerular filtration rate (eGFR) was calculated using the Modification of Diet in Renal Diseases (MDRD) equation [14] as this was the equation in use in all laboratories during the follow-up period. % Calculated reaction frequency (cRF, defined as percentage of HLA incompatible donors from a pool of 10,000 blood group identical UK donors) was utilized as a crude marker of HLA sensitization [15], along with individual data on donor specific antibodies.

All statistical analysis was carried out using GraphPad Prism 8 (GraphPad Software). Mann-Whitney U tests and Chi-squared were used for between-group differences. Log-rank analyses of Kaplan-Meier curves were used to compare patient and death-censored graft survival. The clinical and research activities being reported are consistent with the Principles of the Declaration of Istanbul as outlined in the ‘Declaration of Istanbul on Organ Trafficking and Transplant Tourism’.

## Results

Data were available for 34 SCD patients who underwent renal transplantation between 1997 and 2017, across the 6 London Units, no patients were excluded from the analysis. 16 transplants resulted from donation after brain death (DBD), 6 from donation after circulatory death (DCD), and 12 were from live donors (LD) (including 1 in the UK Paired and Pooled Scheme). Median follow-up time was 53 months (range 1 to 240 months) and no patients were lost to follow up.

18/34 patients (53%) were male. 5/34 (15%) had an HbSC genotype; the remaining 29 (85%) had an HbSS phenotype. The cause of ESRD was biopsy proven SCN in 20 patients (59%, 18 HbSS, 2 HbSC)), presumed SCN (not biopsied) in 9 patients (26%, 6 HbSS, 3HbSC), lupus nephritis in 1 patient (3%), AA Amyloid in 1 patient (3%), and unknown (small kidneys) in 2 patients (6%). None of the transplants were pre-emptive; median time spent on dialysis prior to transplantation was 39 months (range 2 to 177 months). Median age at the time of transplantation was 36 years (range 23–52 years). 11/34 patients (32%) had at least one sensitizing event prior to transplantation (previous pregnancy in 8, previous kidney transplant in 3 patients). None of the recipients had pre-formed donor-specific antibodies to HLA.

29/34 patients (85%) received basiliximab induction followed by maintenance immunosuppression with a calcineurin inhibitor (ciclosporin or tacrolimus), azathioprine/mycophenolate mofetil (MMF) and corticosteroids. 1 patient received anti-thymocyte globulin (ATG) induction and maintenance immunosuppression with ciclosporin and MMF, and 1 patient received alemtuzumab and tacrolimus monotherapy. The induction agent was unknown in 3 patients. The calcineurin inhibitor used was tacrolimus in 21/34 patients (62%) and ciclosporin in 13/34 patients (38%). 20 of the 22 deceased donor recipients (90%) experienced delayed graft function (DGF, defined as the need for dialysis after transplantation), compared to 2 of the 12 living donor recipients (17%).

Overall 1, 5- and 10-year patient survival was 91%, 72% and 33% respectively, with death-censored graft survival at 87%, 61% and 19%. A total of 10 patients died during the follow-up period, 4 with a functioning graft (3 from sepsis and 1 from a SCD-related cerebrovascular accident), and 6 subsequent to graft failure (cause of death not known). Of the 3 deaths from sepsis, one patient died from intra-abdominal sepsis and 2 patients died from sepsis of uncertain origin.

Of the 13 patients who lost their grafts, the causes were recorded as: recurrent SCN in 5 patients, rejection in 5 patients, primary non-function (PNF, defined as the permanent loss of allograft function immediately after transplantation) in 2 patients and graft thrombosis within one month in 1 patient. The overall incidence of biopsy-proven rejection was 38%.

### EBT versus no EBT

20/34 patients (59%) received automated EBT for an extended period of time following the renal transplant with the aim of reducing sickle complications, maintaining Hb >90g/l and HbS< 30%. 3 of these 20 patients (15%) were established on EBT prior to transplantation, 8 patients (40%) commenced EBT at the time of transplantation, and 9 patients (45%) started EBT after transplantation. EBT commenced between 31 months prior to transplant and 48 months post-transplant. The time spent on an EBT program post-transplantation ranged from 4–124 months (median 48 months). 17/20 patients continued for the remaining follow-up time, 3/20 patients discontinued regular EBT during the follow-up period (1 patient due to non-compliance, no data on the other 2 patients).

The median number of blood units transfused per year in patients receiving EBT was 37 units (range 20 to 120 units), as compared to 8 units per year (range 2 to 17 units) in patients on top up transfusion (regular or intermittent) (p = 0.0004). Median HbS% at the time of transplantation was 17% in those treated with EBT (range 4.8–20.3%) and 31.4% in those not treated with EBT (range 5.6–72%) (p = 0.049).

Overall the patient characteristics of EBT and no EBT groups were comparable (Table 1), apart from the year of transplantation; those receiving EBT had a median year of transplantation of 2012 compared to 2005 for patients not undergoing EBT. In line with this finding and with changing trends in immunosuppression use over time, tacrolimus was the CNI used in the majority of the EBT group while ciclosporin was more commonly used in the no EBT group.

**Table 1:** Patient characteristics in the population as a whole and the two groups (those receiving EBT and those not receiving EBT).

Patient characteristic		All patients (n = 34)	EBT (n = 20)	No EBT (n = 14)	p value
Female gender		16 (47%)	8 (40%)	8 (57%)	0.32
SCD genotype	HbSS	29 (85%)	17 (85%)	12 (86%)	0.99
	HbSC	5 (15%)	3 (15%)	2 (14%)	0.99
Median age at transplantation in years (range)		36 (23–52)	39.5 (23–52)	34 (25–45)	0.99
Median year of transplantation (range)		2010 (1996–2017)	2012 (2006–2017)	2005 (1996–2017)	0.01
Type of Tx	LD	12 (35%)	7 (35%)	5 (36%)	0.37
	DBD	16 (47%)	8 (40%)	8 (57%)	
	DCD	6 (18%)	5 (25%)	1 (7%)	
Median Cold Ischaemic Time in hours (range)		12 (2.8–19)	12.75 (2.8–17)	12 (3.5–19)	0.94
Mean HLA mismatches	A	0.90	1.05	0.71	0.33
	B	1.08	1.20	0.86	0.32
	DR	0.59	0.65	0.43	0.62
Immunosuppression:					
Induction	Basiliximab	29 (85%)	18 (90%)	11 (79%)	
	ATG	1 (3%)	0	1 (7%)	
	Alemtuzumab	1 (3%)	1 (5%)	0	
	Unknown	3 (9%)	1 (5%)	2 (14%)	
Maintenance	Tacrolimus	21 (62%)	15 (75%)	6 (43%)	0.06
	Ciclosporin	13 (38%)	5 (25%)	8 (57%)	
	Prednisolone	31 (91%)	18 (90%)	13 (93%)	
	Azathioprine	5 (15%)	1 (5%)	4 (29%)	
	MMF	25 (74%)	16 (80%)	9 (64%)	

**Fig 1:**
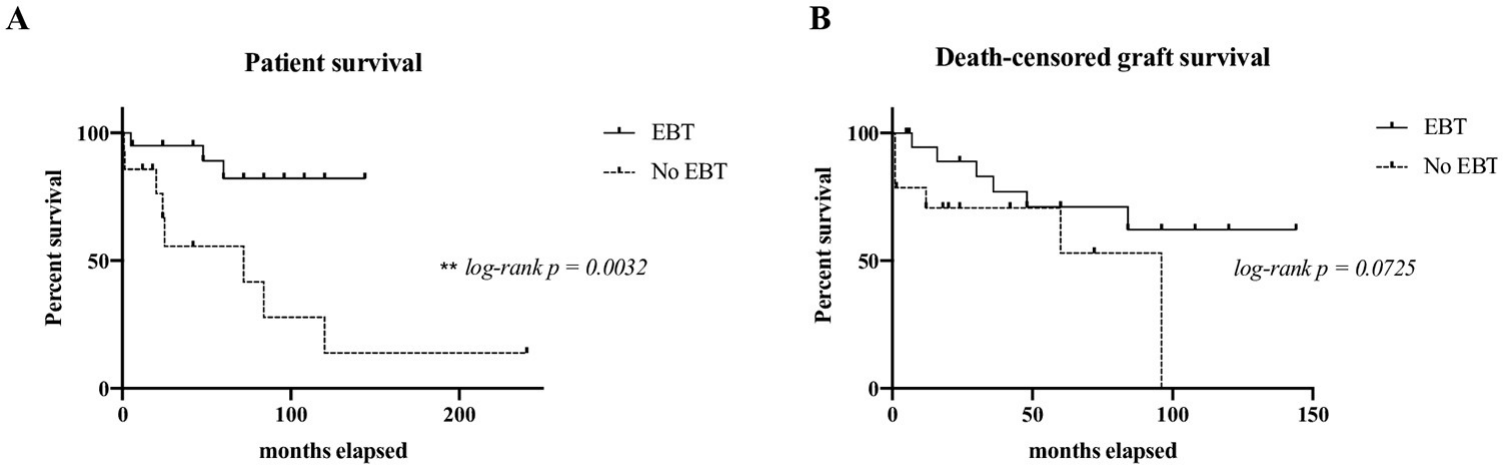
Kaplan -Meier survival curves comparing (A) patient survival, and (B) death-censored graft survival with and without EBT.

**Fig 2:**
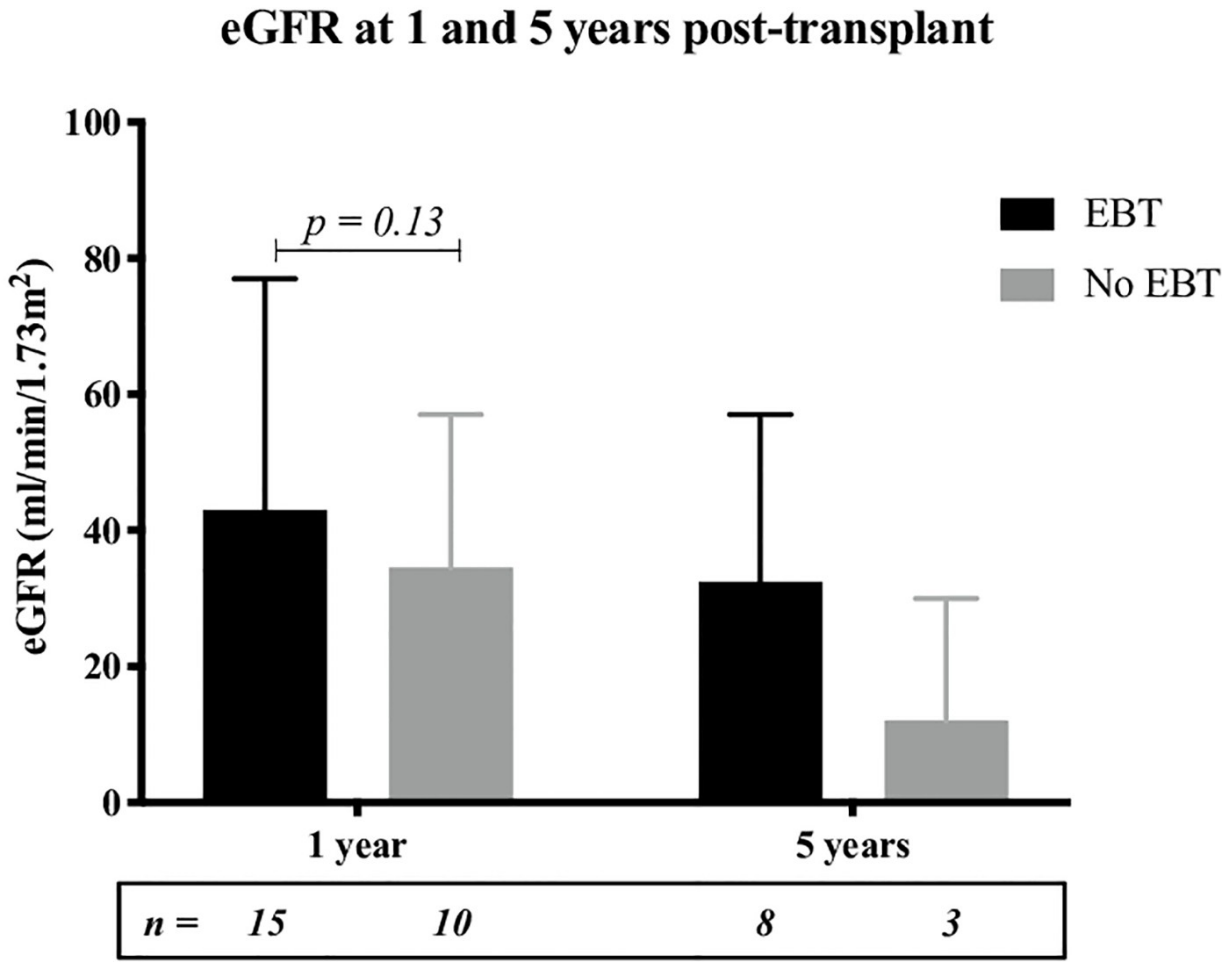
Median eGFR (calculated by MDRD/ml/min) at 1 and 5 years post transplant with and without EBT. Error bars represent range.

Patient survival, death censored graft survival and graft function (as estimated by MDRD eGFR) were superior at all time-points post-transplant in those on an EBT program compared to those not on an EBT program (Table 2, Figs 1 & 2). The difference in patient survival was particularly pronounced, reaching statistical significance with a confidence interval of 95% (p = 0.0032).

**Table 2:** Patient and death-censored graft survival (in %), and median eGFR (in ml/min/1.73m^2^) at 1, 5 and 10 years post-transplantation in those on an EBT programme (EBT) compared to those not on an EBT programme (No EBT), alongside UK data for DBD, DCD and LD transplants (Source: NHSBT^17^). Numbers of patients available at each follow-up included below each row of data (n).

		EBT (n = 20)	No EBT (n = 14)	UK average DBD/DCD/LD (2004–2006 cohort)^17^
**1 year**	Patient survival	95	86	97/95/99
	*n*	*19*	*12*	
	Graft survival	94	77	93/94/96
	*n*	*18*	*10*	
	Median eGFR	45	33	53
**5 years**	Patient survival	88	44	90/86/96
	*n*	*13*	*5*	
	Graft survival	67	50	85/87/92
	*n*	*10*	*4*	
	Median eGFR	34	12	
**10 years**	Patient survival	60	29	76/72/91
	*n*	*3*	*2*	
	Graft survival	33	0	76/76/82
	*n*	*3*	*0*	
	Median eGFR	29	n/a	

In the analysis of graft-specific outcomes, 2 patients with PNF were removed (both from the no EBT group). The incidence of biopsy-proven rejection in those on an EBT program was 5/20 (25%), as compared to 7/12 (58%) (p = 0.06) not on an EBT program (Table 3).

**Table 3:** Sensitization and graft specific outcomes in those on an EBT programme compared with those not on an EBT programme.

	EBT (n = 20)	No EBT (n = 14)	p value
**Median cRF**	34%	71%	0.07
**Incidence of DSA**	20%	21%	0.92
**Incidence of rejection**	25%	58%	0.06
**Incidence of recurrent SCN**	20%	50%	0.08

**Fig 3:**
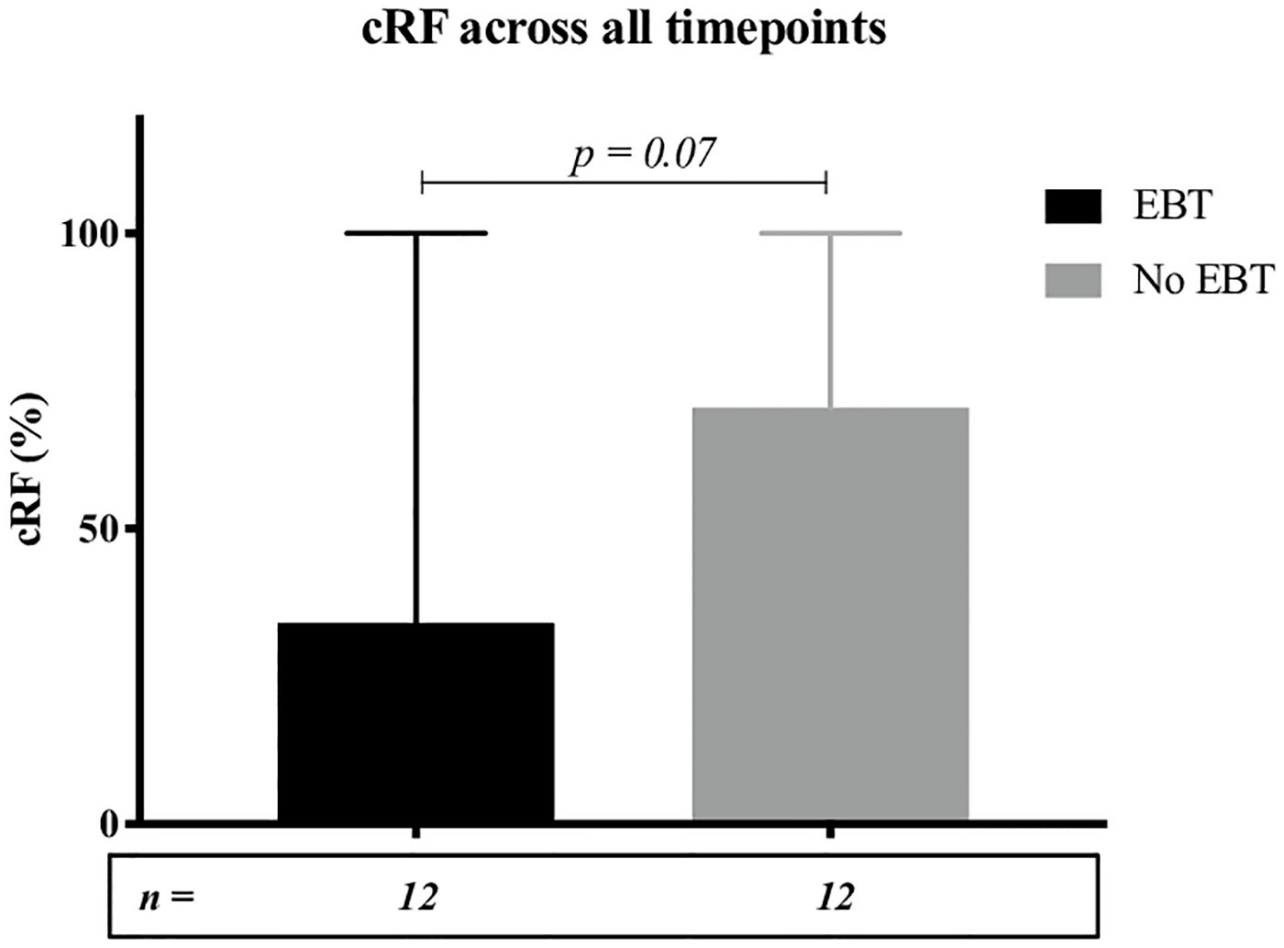
Median cRF(%) across all timepoints with and without EBT. Error bars represent range.

Median calculated reaction frequency (cRF) across all time points (pre- and post-transplantation) was 34% in patients on an EBT program and 71% in patients not on an EBT program (p = 0.07) (Fig 3 & Table 3). 4/20 patients (20%) on an EBT program developed *de novo* donor specific antibodies (DSAs). 3/14 patients (21%) not on an EBT program developed *de novo* DSAs (Table 4).

**Table 4:** Donor-Specific Antibody (DSA) data, including Mean Fluorescence Intensity (MFI) where known, timing and associated graft outcomes, in those on an EBT programme compared with those not on an EBT programme.

	Patient	Details of DSA (MFI)	Time to appearance (months)	?associated with rejection/graft loss
**EBT**	1	A2 (2297)	108	No
	2	B8, C7, DQ5 (no MFI data)	unknown	No
	3	A2, A68, A69 (no MFI data)	unknown	No
	4	B51 (weak) C16 (no MFI data)	<1	No
**No EBT**	1	B41, B49, B50, B42 (no MFI data)	unknown	No
	2	A24(2346–5685), B8(1188–1918), DR0103(1423–2455), DQ5(4372–6532)	30	Yes (CAMR at 42 months, graft loss due to chronic rejection at 84 months)
	3	A3(1219), A29(8828), B8(2768), DR1 (1012)	<1	Yes, graft loss

CAMR = Chronic Antibody-Mediated Rejection.

**Fig 4:**
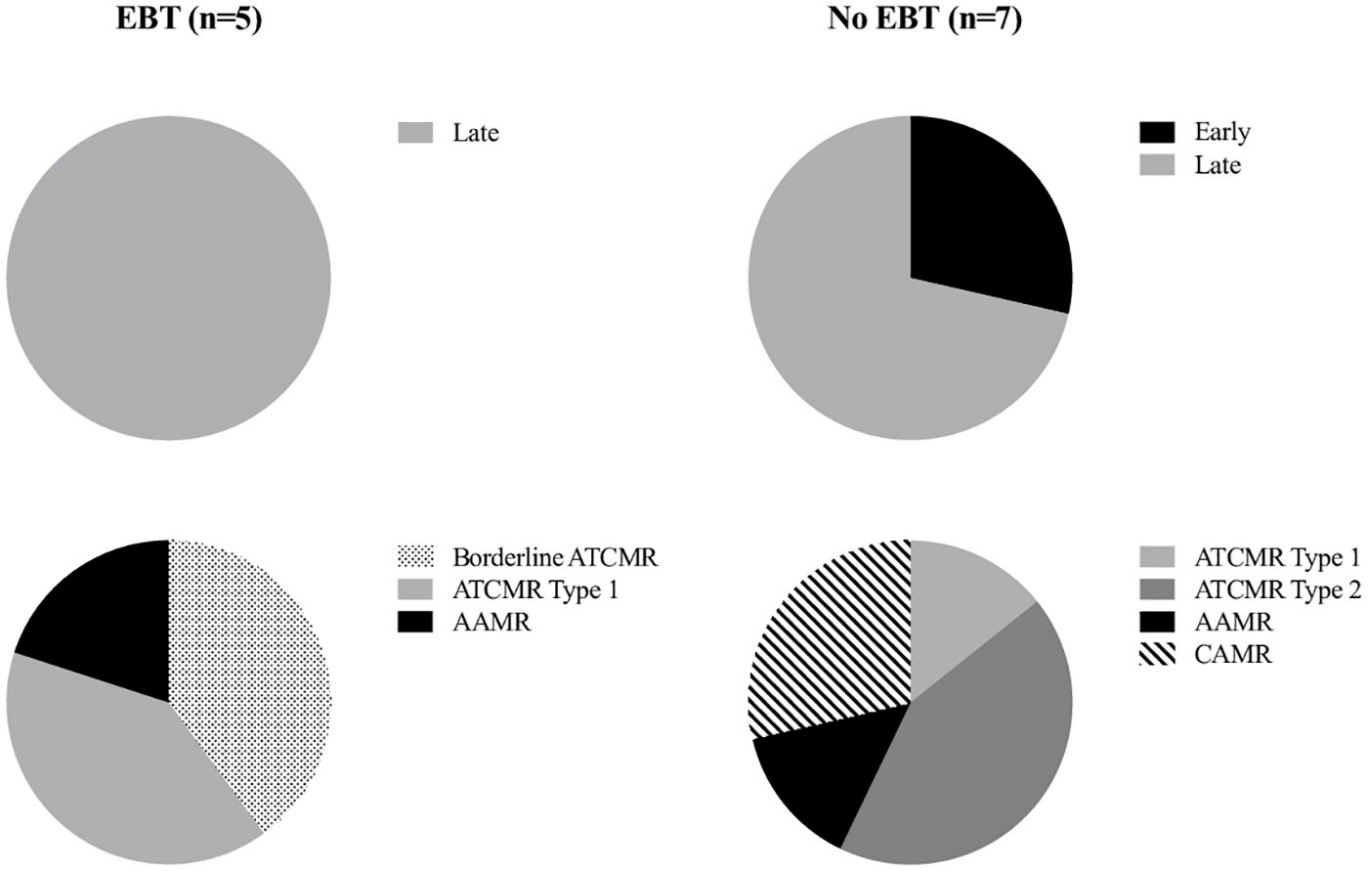
Details of rejection timing (early, < 3 months after transplantation, vs late, > three months after transplantation) and Banff Classification compared between EBT and no EBT groups. ATCMR = Acute T cell-mediated rejection, AAMR = Acute antibody-mediated rejection, CAMR = Chronic antibody-mediated rejection.

Of the 12 patients who experienced biopsy-proved rejection, 2 patients (both in the no EBT group) experienced early rejection (within 3 months of transplantation), the remaining 10 patients experiencing late rejection (more than 3 months after transplantation), with a median time from transplantation of 13.5 months (range 1 to 42 months). In the EBT group 2 patients experienced borderline changes (suspicious for acute cellular rejection), 2 patients experienced acute cellular rejection (Banff^16^ Type 1B in both cases), and 1 patient experienced acute antibody-mediated rejection. In the no EBT group, 4 patients experienced acute cellular rejection (1 with Banff Type 1A, 3 with Banff Type 2 A or B), 2 patients experienced acute antibody-mediated rejection and 1 patient experienced chronic antibody-mediated rejection (Fig 4). All cases of biopsy-proven rejection were treated with pulsed intravenous methylprednisolone (3 doses of 0.5-1g), and all patients had optimization of their maintenance immunosuppression (including a switch from ciclosporin to tacrolimus, and an increase in, or restart of their MMF). One patient (in the no EBT group) received anti-thymocyte globulin (ATG) for vascular rejection (Banff Type 2) [16], and died from intra-abdominal sepsis two weeks later. No other monoclonal antibodies, plasma exchange or intravenous immunoglobulin were used in the treatment of rejection.

Recurrence of SCN was seen in 4/20 patients (20%) on an EBT program, as compared to 6/12 patients (50%) not on an EBT program (p = 0.08).

### Hydroxycarbamide treatment

2/34 patients (6%), not receiving EBT, were treated before and after transplantation with hydroxycarbamide.

The first received a DBD transplant in 1996 and was started on hydroxycarbamide due to recurrent sickle cell crises post-transplantation. This patient had an eGFR of 27ml/min/1.73m^2^ at 1 year and 30ml/min/1.73m^2^ at 5 years and died of sepsis/sickle cell crisis with a functioning graft 72 months post-transplantation. The second received a LD transplant in 2016 and was treated with hydoxycarbamide until 6 months pre-transplantation, and again from 10 months post-transplantation. This patient remains under follow-up with a functioning graft and an eGFR of 42ml/min/1.73m^2^ at 1 year.

## Discussion

To date this is one of the largest case series to report on the outcomes of adult SCD patients undergoing renal transplantation and the only one to have comparative data for those treated with or without automated EBT. In this cohort, the most striking finding is the significantly increased patient survival in those treated with automated EBT post transplantation. In addition, graft survival and function were superior in SCD patients receiving EBT, with lower rates of SCN recurrence in the graft and no increase in HLA sensitization, DSA formation or rejection, despite an increased transfusion burden.

Overall patient and graft survival post-transplantation were inferior to contemporaneous UK data in the ESRD population as a whole [17] but this is not a new finding, and it is well-established that transplantation carries with it a better prognosis than dialysis in SCD patients with ESRD [9, 10].

Overall rejection rates were high at 38% in this population, and while transfusion-related HLA sensitization may well play a part in this, SCD-related tissue hypoxia and consequent immunoactivation is also a possible contributing factor, particularly in recipients of deceased donor kidneys. Crucially, it would appear that the risk of rejection is no higher with the increased transfusion burden associated with regular automated EBT programs, as compared to patients who received intermittent top-up transfusion. Indeed, there was a trend towards lower rejection rates in those treated with EBT, although it is difficult to draw definitive conclusions from this in light of the differences between the groups in year of transplantation and CNI choice. It is worth noting that all blood for patients with SCD in the UK is extended phenotype matching and is full matched for ABO, Rh and Kell type, to reduce the development of red cell allo-antibodies.

While the LD vs DD population was comparable between the 2 groups, there was a 7-year difference in the median date of transplantation. While long-term outcomes did not change to such a dramatic effect in follow-up data from UK-wide cohorts transplanted from 2005 to 2012, it is likely at least some of the survival benefit seen in the EBT group may simply reflect improved care in more recent times [18], particularly as the EBT group had a higher proportion of patients on tacrolimus (as opposed to ciclosporin) than the no EBT group. This is clearly a major limitation to the study and diminishes the extent to which conclusions can be drawn. However, it is clear that outcomes using modern immunosuppression and EBT are far superior to historical therapies, and as such we would speculate that modern immunosuppression is sufficiently powerful to protect from the potentially harmful effects of HLA sensitization in patients receiving EBT, potentially justifying the future use of EBT without historical fears.

Although blood transfusion pre-transplantation is generally avoided due to the risk of sensitization, there remains debate on its impact [12]. The effect of transfusion post transplantation (with a functioning graft) is less well studied but is thought to increase the risk of developing a DSA [19]. Interestingly, the incidence of *de novo* DSA development in this cohort was no higher with EBT than without it. Furthermore, the development of *de novo* DSAs in the post-transplant period was not associated with rejection or graft loss in any of the patients on an EBT program. More work is needed on the impact of large volume and regular exchange transfusions on HLA sensitization, and clearly this finding is also confounded by the difference in date of transplantation and consequent immunosuppression practice between groups, but the data available from this cohort is reassuring.

Acute vaso-occlusive crises (VOC) are reported as rare in patients receiving dialysis but the frequency increases post renal transplantation, possibly due to a rise in endogenous erythropoietin release [20, 11]. Although we did not collect data on VOC in our patients, VOCs are less severe in patients on automated EBT programs [21], and whilst this benefit may also be seen with regular simple transfusion, automated EBT will lead to an improved control of HbS% and reduced iron overload.

Hydroxycarbamide (HC, also known as hydroxyurea) is a cytotoxic, antimetabolite approved for use in SCD. Although it has pleotropic effects, it primarily acts to increase levels of fetal haemoglobin which dilute the levels of HbS and reduce risk of polymerization. Clinical benefits include lower rates of pain, acute chest syndrome and need for blood transfusion. Long-term usage has been associated with improved growth and development in children and reduced overall mortality and morbidity in adults [22, 23]. While there is no conclusive evidence that it slows progression of CKD in patients with SCD, there is observational data which suggest it may be of benefit in both adults and children by reducing hyperfiltration and albuminuria [24, 25, 26]. Despite this, hydroxycarbamide was not widely used amongst our patients post renal transplantation. This it may be due to fears that it will increase the risk of myelosuppression with concomitant use of antiproliferative immunosuppressive agents, and it must be dose-reduced at low GFRs. However, its disease modifying effects may mean it is a safe and effective alternative to post transplant exchange transfusion and further studies are needed to investigate this.

Sepsis is a significant concern in this population, with 3 out of the 4 deaths with a functioning graft being attributed to sepsis. Further studies are required to investigate the optimum immunosuppression regimen in these patients who due to their SCD have underlying defects in immune function.

The current study has a number of limitations, in addition to the potentially confounding effect of time and changing immunosuppression practices on transplant outcomes in the two groups as already discussed. There were missing data in all categories collected from across all units, which has limited the extent to which conclusions can be drawn. The data were collected retrospectively and are observational in nature so no definitive conclusions can be drawn regarding the causative nature of any associations found. Furthermore, it is possible that patients on EBT programs were more motivated and engaged with their medical care and therefore less likely to suffer the consequences of non-adherence, a well-recognized risk factor for poor outcomes in transplantation [27].

Data were not collected on the effectiveness of EBT at preventing VOC or other complications of SCD, on complications associated with vascular access or on the impact of regular EBT on total body iron. Data were also not collected on co-morbidities in order to better assess the equivalence of the two groups for comparison.

In conclusion, kidney transplantation in SCD patients with ESRD is high risk, but it is well-recognized that outcomes are superior to patients who remain on dialysis [9, 10]. Despite significant limitations our data suggest long-term EBT post renal transplant is effective and safe, with improvement in graft and patient outcomes and no increase in antibody formation. We believe that post-transplant EBT programs should be adopted more widely when transplanting SCD patients, but in order to more rigorously demonstrate this prospective studies are needed. Further evidence is also needed on the role of EBT pre-transplantation. Performing EBT immediately pre-operatively is probably the best approach but this may prove difficult in units that do not have automated EBT available at short notice when transplantation is imminent. We would suggest that a safe alternative is to commence automated EBT in patients on dialysis who have been placed on the deceased donor waiting list to ensure they are prepared when a kidney is offered.

We believe a consensus needs to be sought to determine the optimal time to start EBT in the peri-transplant period for every patient with SCD approaching ESRD and to achieve this, it is vital for haematologists and nephrologists to work closely together. In addition, more work needs to be done to assess the resource requirements and financial impact of a policy including EBT for all SCD patients undergoing kidney transplantation, although we would speculate that any increase in costs associated with EBT would be more than balanced by the cost saving of transplantation versus dialysis, and the potential increased productivity of what is predominantly a working-age population.

## Supporting information

S1 File London Hospitals contributing data to this study. (DOCX)
