# Supplementary material for: Outcomes following kidney transplantation in patients with sickle cell disease: The impact of automated exchange blood transfusion
Source: PLoS One. 2020 Aug 13;15(8):e0236998. doi: 10.1371/journal.pone.0236998 (PMC7425908; doi:10.1371/journal.pone.0236998)
Supplement: S1 File — (DOCX) [file pone.0236998.s001.docx]

**London Hospitals contributing data to this study**

King's College Hospital NHS Foundation Trust

Guy's and St Thomas' NHS Foundation Trust

Royal Free London NHS Foundation Trust
Epsom and St Helier University Hospitals NHS Trust

Imperial College Healthcare NHS Trust

Barts Health NHS Trust
